# Hormonal Influences on Cognitive Function

**DOI:** 10.21315/mjms2018.25.4.3

**Published:** 2018-08-30

**Authors:** Siti Atiyah Ali, Tahamina Begum, Faruque Reza

**Affiliations:** Department of Neurosciences, School of Medical Sciences, Health Campus, Universiti Sains Malaysia, 16150 Kubang Kerian, Kelantan, Malaysia

**Keywords:** hormones, cognitive functions, neuronal network

## Abstract

This article examines how hormonal changes may affect the neuronal networking and mechanisms of cognitive function. Hormones are the chemical regulators of the human body and function critically to maintain various processes, such as growth, emotions and even cognition. Numerous studies have examined the relationship between hormonal effects and cognitive function; these studies have investigated different factors, such as aging, pregnancy, post-natal states, emotions and stress. Different types of hormones produce different outcomes for the human body and mind. Hormones may also contribute to both positive and negative outcomes, depending on whether the hormone levels are too low or too high. To investigate the hormonal effects on cognitive function, the sources of localisation must be localised, so that the neuronal network can be realised. Furthermore, cognitive function does not rely on a specific brain region but is determined by the neuronal network interactions. Thus, it is worthwhile to know the neural mechanisms behind cognitive functions that are affected by hormones.

## Cognitive Function

Cognitive function refers to the brain’s ability to process information about the world and includes thinking, memory, recall, mental flexibility, problem solving and learning (1, 2). Without all these functions operating properly, people can display abnormalities that may be diagnosed as diseases or disorders. Some examples of cognitive impairment are Alzheimer’s disease, dementia and neuropsychiatric disorders (2). Cognitive impairment can be caused by numerous factors (i.e., genetics, aging, lifestyle, environment), but surprisingly, several studies have found that hormonal fluctuations are one of the key factors that lead to defects in cognitive function (3, 4, 5). Hormones are modulated from the beginning of life in utero, as the differentiations of gender begin in utero (6, 7). The sexual/gonadal hormones such as androgen, testosterone, estrogen and progesterone are continuously produced by gonads and are regulated throughout adolescence and adulthood (8).

Apart from sexual hormones, which are commonly associated with cognitive function, a stress hormone such as cortisol is in the class of the glucocorticoid hormone (5). However, hormonal levels may not remain well-maintained when certain triggers cross their pathways, such as aging and pregnancy. “It has also been established that gonadal hormones, including estradiol, can act at membrane receptors to activate intra-cellular signaling mechanisms which alter cellular function” (8, 9, 10). Apart from the synthesis of certain hormones (i.e., neurosteroids/gonadal hormones) in the brain areas, estradiol may increase the chance of altering cognition, emotions (11) and other neural factors (12). This review article discusses the literature on the hormonal relationship to neural mechanisms in cognitive function, along with related findings in neuroscience research.

Cognitive function deficit is identified through a neuropsychological assessment that comprises language, attention, conceptual reasoning, memory and processing speed (13, 14). The sex steroidal hormones mechanism begins once these hormones bind to the target tissue and translocate the hormone-receptor complex into the nucleus. Then, the complex structure interacts with chromatin inside the nucleus and changes the DNA (deoxyribonucleic acid) structure of chromatin (15) for the gene transcription process. The steroid hormones interact with the chromatin, causing an increase in ribonucleic acid (RNA) synthesis, which allows the synthesis of protein (15).

Estrogen and progesterone regulate physiology and reproductive behaviour. These hormones are believed to be responsible for activating the estrogen receptor (ER), which leads to the expression of genes, including the progesterone gene (10, 16). Estrogen induces the progesterone receptor (PR), functioning as a transcriptional mediator and regulating the transcription of target genes (17). The time taken to activate and terminate reproductive behaviour is comparable to the time estrogen takes to increase and decrease PR in the hypothalamus. However, the progesterone and estrogen pathway mechanisms are not well-defined in many aspects, particularly these hormones’ reactions towards tissue (18). Progesterone is the main hormone regulated at three different stages: the maintenance of the uterus; the development of oocytes (the mammary gland for milk production); and sexually responsive behaviour in the brain (18, 19, 20).

Estrogen has been investigated as a hormone with the potential ability to act as a neuro-protector, which may selectively benefit certain cognitive tasks such as memory-forming mediated by the hippocampus and frontal lobe (21). Estrogen hormone therapy is being selectively suggested for menopausal women who are experiencing a decline in cognitive function, for this type of therapy is believed to reduce the risk of getting dementia (22) and Alzheimer’s disease. Neuroimaging findings lend support to hormone therapy’s ability to act as a neuroprotector for menopausal women (21), which can prevent the aging-related risk of brain atrophy, specifically in the frontal lobe and hippocampal cognitive area (21, 23). Another benefit of the maternal hormones (estrogen and progesterone) is their ability to stimulate neuron outgrowth, synaptogenesis and dendritic branching in neuroplasticity (24).

Marss et al. (25) claim that maternal hormones for pregnant women have an impact on the latter’s prefrontal and hippocampal function tasks, such as attention, working memory and executive functions. Marss et al.’s (25) study is one of the most comprehensive and most recent studies that has investigated the relationship between hormones and cognitive function. Marss et al. (25) investigated the levels of six different maternal hormones present in saliva samples: progesterone, dehydroepiandrosterone sulfate (DHEAS), testosterone, estrone, estradiol and estriol. These hormone levels were then correlated with a range of cognitive tests that assessed verbal memory, speed processing and attention (i.e., the California Verbal Learning test II (CVLT-II), the Paced Auditory Serial Attention Test (PASAT), the Rey Complex Figure Test (CFT), the Finger Tapping Test (FTT), the Purdue Pegboard, the Verbal Fluency Test, the CFT-recall and the Design Fluency Test). The correlation tests of these variables revealed positive correlations between overly high/overly low hormones with reduced verbal memory, attention and processing skills during pregnancy, except for DHEAS, where no correlation was found (25). Women in late pregnancy may also experience cognitive difficulties due to significantly high hormone levels (26). However, the state of deficit in cognitive function during pregnancy does not have a high impact on daily life, with most studies finding that pregnant women tend to have normal or mild cognitive impairment (27, 28, 29). In contrast, a study by Christensen et al. (30) found a different result; in comparing the cognitive and executive functions between a group of pregnant women and a group of women in early motherhood, Christensen et al. found no positive findings to associate pregnancy and motherhood with a persistent decline in cognitive function (30). However, their study may have been limited due to the restrictions of the tests that were conducted with the neuropsychology test alone (i.e., cognitive speed, working memory, immediate recall memory), such as the Symbol-Digit Modalities Test (SDMT), the Wechsler Memory Scale and the California Verbal Learning Test, all of which do not consist of an objective test, such as the Electroencephalography (EEG), the Event Related Potential (ERP) or the functional Magnetic Resonance Imaging (fMRI). Furthermore, Christensen et al. acknowledge that the subjects’ variations due to their different backgrounds may have affected the research findings. Due to these limitations, extended research is needed to clarify these findings (30).

Nevertheless, the use of fMRI in several studies has indicated that some changes are observed in brain reactivity across the menstrual cycle, and these changes are mostly prominent in increased amygdala reactivity in the luteal phase (31). The amygdala functions as the governor of the independent memory system, together with the hippocampal complex, which interacts when motion meets memory (32). Impaired cognitive function during the menstrual cycle has been reported by Bayer et al. (33), where there was an impairment in the cognition of negatively valence stimuli in the mid luteal menstrual phase, with “no differences in behavior which have been reported in the longitudinal fMRI studies evaluating menstrual cycle effects” (33). Parallel findings on the change of lateralisation in functional cerebral asymmetries (FCAs) during women’s menstrual cycle indicate the inhibition of the dominant to non-dominant hemisphere, and this could be influenced by hormonal changes during menstruation (34). This finding has been contradicted by a recent study by (35), who found that the menstrual cycle does not cause cognitive abilities to deteriorate. However, the results observed during the first cycle in this study were not replicated in the second cycle, which may suggest the probability of false-positive attributes by random variation and systematic biases (35).

Some studies have found little connection between hormones and cognitive function. For instance, Koyama et al. (36) conducted a prospective cohort study, following up 3,044 women over a span of 23 years. The study consisted of three different phases of data collection: the blood plasma collection phase; neurologic testing; and inquired cognition of the later age phase. There were seven plasma hormones plasma tested: estrone, estrone sulfate, estradiol, androstenedione, testosterone, dehydroepiandrosterone (DHEA) and DHEAS. Tests used in the second phase of neurologic testing were the Telephone Interview of Cognitive Status (TICS) (verbal memory recalled); a telephone version of the Mini Mental State Examination (MMSE); the East Boston Memory Test (which assesses immediate and delayed recalls); the category fluency test (for measuring semantic memory); and the Backward Digit Span (working memory and processing speed) (36). This study revealed a non-significant relation between plasma levels of most maternal hormones and neuropsychology test performances, with the exception of plasma estrone, which showed a small positive association between higher plasma estrone and better cognitive performance, supporting the role of the neuro-protective agent of estrogen (36). Other than that, sex steroidal hormones (i.e., estradiol, estrone, parity, testosterone, androgens, estrogens and progesterone) are believed capable of inducing neurogenesis in the hippocampus for both males and females, as sex steroidal hormones have diverse targets in the central nervous system (CNS) (12). The hippocampus works with other regions of the brain and does not function as a single structure; it is functionally dissociated along the dorso-ventral axis (12, 37). Dispersed maternal hormone receptors are localised in the hippocampus, hypothalamus and amygdala area, and these receptors’ reactions can be mediated by neurogenesis and neuroplasticity in the brain (24) through multiple intracellular signalling pathways, including MAPK/ERK and the Akt pathway, which are involved in the cell survival process (24, 38). In addition, it is believed that impairment in this stage of the process is one of the factors in depression, mood swings, impaired working memory and executive controls (24, 39).

In the case of over-secretion of stress hormones, the class of glucocorticoids have been identified as cofactors that cause impairment of memory, but in certain ways, they can enhance memory performance (5). In the course of surviving enormous daily life changes, people are prone to many types of stress inducers, and in order to cope with stress, several neural processes become activated to develop mental and emotional coping strategies. The prefrontal cortex is responsible for making decisions about staying calm and thinking of solutions, while the amygdala generates emotional responses (i.e., anxiety, anger, happiness and fear). These structures are linked to the hippocampus in order to retrieve the relevant emotional memory so that a proper action or response can be generated (40, 41). High levels (40) and low levels (42) of glucocorticoids can impair the ability of memory due to altering the brain circuit responsible for consolidation of memory and memory storage. Conversely, moderate levels of glucocorticoid (42) and epinephrine (43, 44, 45) may enhance memory consolidation in the hippocampus. The hippocampus is highly sensitive to changing levels of glucocorticoids, and fluctuating levels of this hormone are highly associated with hippocampal atrophy (46), Alzheimer’s disease (47) and major depressive disorders (40, 48). In short, intense stress levels do appear to have a negative impact on memory ability.

Hormonal disturbance of cognitive function and emotions is commonly associated with women, as they have monthly hormonal regulation during the menstrual cycle and during pregnancy. However, hormonal disturbances can also affect men (11). Studies regarding hormones’ impact on male cognitive function is limited, as many previous studies focused on maternal hormones. In line with this scarcity of studies, there are not enough supportive findings to confidently claim hormonal influences on different cognitive functions (i.e., verbal fluency, perceptual speed and memory) among males and females (49, 50). However, a recent study by Mahmoud et al. (12) has suggested that the hormonal influence on cognitive function between genders might be relevant due to the different abilities of sex hormones to induce neurogenesis in the hippocampus (12). Berenbaum et al. (49) claim that sexual differentiation related to hormones takes place during the early prenatal and early postnatal phases, in which the high level of androgen induces the development of spatial ability and learning abilities (49). A low level of testosterone across ages can reduce cognitive function (51, 52). A review of scientific evidence about the differences of hormonal effects towards different genders was undertaken by Torres et al. (50), who concluded that female subjects perform better in verbal fluency, perceptual speed tasks, fine motor skills, verbal memory and verbal learning (50, 53). Males, however, are better in visuospatial ability, mathematical problem solving and visual memory (50). A recent review article by Ngun et al. (54) mentioned that gonadal hormones might be influenced by genetic factors, whereby the encoded genes in sex chromosomes act directly on the brain to influence neural development, and neural spatial explain the different sexual behaviour between different genders (54). However, these findings cannot be conclusive in determining the effect of hormonal differences, due to limited information about the types or level of hormones that are being studied among male and female subjects. Nevertheless, these scientific reviews by Torres et al. (50) and Ngun et al. (54) provide an insight into the view that hormonal effects may be manifested differently between males and females.

## Hormonal Influences and Their Localisation

Integrated hormonal effects and their localising were mainly done by conducting studies on animals, as this was more practical and provided the foundation for theorising about human mental processes. Thus, various studies about hormones and behaviour/cognitive function were conducted among animals (although in some studies, experiments used human studies). Hormonal levels combined with subjective tests (neuropsychology tests) and objective tests (ERP, fMRI) were assessed on human subjects, as these combinations are more practical, precise, less risky and safe for human subjects.

Lupien et al. (5) investigated the part of the brain associated with cognition and stress hormones. Glucocorticoids’ (stress hormones’) receptors are localised at brain structures that are known for memory and learning processes: the hippocampus, amygdala and frontal lobes. The stress hormones are closely related to the mechanism of fear-learning tasks in animals, which is essential for species survival (5). As mentioned in Lupien et al.’s review, secretion of stress mediators enhances the encoding of emotionally relevant emotion, explaining the fear of an animal once being exposed to danger/a predator for the second time, as the memory of fear is consolidated as emotions. Table 1 on the next page shows the lists and summaries of the few reviews/studies on cognitive function and their methodologies in explaining the hormonal influences on brain localisation and human/animal cognition.

## Hormonal System in Humans and Hormonal Influences on Cognitive Functions

The hormonal system (also known as the endocrine system) is important for human well-being, as without these chemical regulators, protein essential for human growth would not be secreted (55). Yet research suggests that hormones might also have negative effects on our brain, especially on cognitive function (56). However, not all hormonal effects are negative, as previous studies have found that certain steroidal/sexual/maternal hormones such as estrogen give neuro-protection in the hippocampus, which protects the brain from effects of aging (57, 58, 59). Estrogen, mostly known as the female sex hormone, is produced by the developing follicles in the ovaries, corpus luteum and placenta (59). Estrogen is manifested and highly expressed in the brain, mainly in the hippocampus area and cerebral cortex, where two different types of estrogen receptors reside in these regions, namely, ER-alpha and ER-beta (59). Numerous studies confirm that estrogen acts as a neuroprotecting agent against aging effects such as dementia; however, the underlying mechanisms for how these hormones protect the brain are still under investigation (59).

Glucocorticoid is a hormone responsible for stress hormones and is released by the activation of the hypothalamic pituitary adrenal (HPA) axis (57), which is closely related to memory retrieval function (40). It has been reported that any dysfunction of the HPA axis leads to high levels of glucocorticoids and causes hippocampal atrophy in Alzheimer’s disease by influencing the deposit of amyloid and tau phosphorylation (40, 58) and increasing the risk of dementia, especially among the elderly (3, 58). These deregulations towards hippocampal neurons may have a negative effect on memory performance (59). In short, induced high levels of stress may affect the HPA axis, thus leading to increased levels of glucocorticoids that might reduce the performance of declarative memory by indirect damage to the hippocampus. High levels of the cortisol hormone (a class of glucocorticoid hormone), induced by chronic stress, leads to overproduction of the myelin sheath and fewer neurons in the hippocampus through the process of oligodendrogenesis (45). In human studies, glucocorticoids were injected in subjects, and subsequent brain activity was measured using an Event Related Potential (ERP) test. The electrical brain activity among the subjects decreased when the latter were exposed to visual stimuli, as the researcher concluded that the glucocorticoids “may have decreased the participants’ ability to attend to the stimuli and thus reflected a state of hypovigilance” (5, 60). Oligondendrogenesis may also damage the structure of white matter and cellular structures (45).

Research findings that have been discussed here have identified that sexual hormones such as estrogen may also play a role in memory, cognition and spatial tasks (61). There are estrogen receptors at multiple sites of brain regions: the hippocampal formation (HF), the amygdala and the cerebral cortex (61). These three regions are responsible for the critical functioning of emotions, memory consolidation and retrieval, and cognitive processes in general. Some studies of pregnant women have related the fluctuations of the sex hormones progesterone and estrogen during pregnancy (61) to their ability to cause attention, memory and executive function deficits, as the hippocampus is sensitive to changes in sexual reproductive hormones (62). The interactions of sex hormones with the brain could be strongly linked (via the neuromodulatory properties of gamma-aminobutyric acid and glutamate receptors) across cerebral hemispheres with cortical areas (63).

The testosterone hormone, which is predominantly higher in males than females, is believed to be one of the sexual hormones that may reduce cognitive performance, as its levels decrease with aging. In young males, spatial ability is highly elevated and is associated with the levels of testosterone that are higher in young males compared to later in life (64–67). This hormone production is stimulated by the gonadotropin-releasing hormone (GnRH) in the hypothalamus and activation of the pituitary gland to secrete certain levels of testosterone into the bloodstream (66). However, the claim of cognitive memory deficits due to testosterone is still debatable (52), as some studies have found that cognition and testosterone levels have a positive linear correlation (67), while some studies have found an inverse correlation (68).

## Conclusion

Hormonal function is important in maintaining the development of human growth and cognitive function. Neuronal networking is still being researched in regard to the exact pathway for how hormonal changes can directly affect cognitive abilities such as memory, thinking, problem solving, spatial ability and even emotion. It does not only affect these negatively, but it may also assist as neuro-protectors, as shown by estrogen hormones. Thus, well-maintained hormones throughout life are crucial to support cognitive abilities for both males and females.

## Figures and Tables

**Table 1:** The methodologies of few researches on hormonal and cognition

No.	Studies/Authors	Methodology	Results/Findings
1.	Estradiol and cognitive function: past, present and future (9)	This paper reviewed the animal and human model in mechanisms of the gonadal hormone, estradiol, and how it influences cognition.	A prominent discussion in this review paper was the mediation of estradiol neural sites in the cerebral cortex, basal forebrain, hippocampus and striatum. This mediation is essential in the higher order of neural cognitive function for memory consolidation. Rodents’ cognition: the organisation of events by estradiol leaves long-lasting imprints that modulate sex hormones differently between male and female rodents, resulting in different cognitive abilities. The male rodents outperformed females on tasks requiring spatial memory in the radial arm maze task (9, 69). Human cognition: estrogen studies indicated possible interactions of the hypothalamic-pituitary gonadal axis (HPG) with the hypothalamic pituitary adrenal (HPA) axis. These HPA interactions activated high stress levels, indicated by the presence of the stress hormone, and can alter gonadal hormones’ function, which can either impair or enhance memory depending on the duration and intensity of stress.
2.	Sex hormones and adult hippocampal neurogenesis: regulation, implications, and potential mechanisms (12)	This review article discussed the modulating roles of sex hormones that regulate hippocampal neurogenesis in males and females, with potential implications for cognitive function and mood regulation. The measurement of neurogenesis was achieved by the assessment of endogenous protein expression (Ki67) as the indication of cell proliferation.	The core discussions revealed that neural stems cells were produced by lateral ventricles that send newly generated cells along the rostral migratory stream to the olfactory bulb and the sub granular zone (SGZ) of the hippocampus of animals, which are influenced by sex hormones. Conversely, human neurogenesis does not occur in the SGZ area but in the hippocampus instead. Throughout the neurogenesis process, the survival of new cells depends on the proliferation, migration and differentiation of newly generated cells into neurons. Hence, this leads to neural organisation and neural sprouting, depending on the level of sex hormones, and eventually influences cognition and memory abilities. These explain the incidence rates of neuropsychiatric disorders such as depression, anxiety and Alzheimer’s disease not being balanced between genders (12).
3	Estrogen and cognitive functions (61)	The authors reviewed the effects of estrogen on behaviour, working memory, cognition and the aging brain.	In animal studies, researchers discovered that there was a predominant localisation of the estrogen receptor (ER) on the plasma membrane of neuritis, soma, dendritic spines and axon terminals. Data showed that estrogen binds and interacts with protein in the mitochondrial membranes and associated its functionality with presynaptic structures. In the end, it controls synaptic transmission, signalling brain function in various ways, including cognition. The widespread prevalence of the ER in the brain influences many neurotransmitters (the GABAnergic system and the serotonegic system) (12). Learning and memory among rodents were improved as estrogen and the ER agonist enhanced the hippocampal formation-dependent memory. The estrogen enhanced performance tasks such as inhibitory avoidance and object recognition (59, 70). This finding is beneficial for research into cognition problems in women, such as Alzheimer’s and dementia, in which the cognitive function reduces with aging, due to low values of estrogen post menopause. In short, estrogen is a kind of hormone that provides protection of cognitive function against aging effects.
4.	Mild auditory cognitive impairment in mid trimester pregnancy (27)	This study was conducted among mid trimester pregnancy females by comparing the auditory attention and executive function with the non-pregnant females as the control by using Event Related Potential (ERP) and five different neuropsychological tests. This study did not investigate a direct correlation between pregnancy hormones and cognitive function. However, it suggests to many researchers that cognitive function differences during pregnancy and non-pregnancy might be related to hormonal influences.	The research finding discovered mild auditory cognitive function among the mid trimester pregnant group, with no impairment of executive function/auditory memory across the neuropsychological tests. The researchers assume that hormonal fluctuations across pregnancy contributed to the differences in cognitive function between groups (27).
